# An Optical Biosensing Strategy Based on Selective Light Absorption and Wavelength Filtering from Chromogenic Reaction

**DOI:** 10.3390/ma11030388

**Published:** 2018-03-06

**Authors:** Hyeong Jin Chun, Yong Duk Han, Yoo Min Park, Ka Ram Kim, Seok Jae Lee, Hyun C. Yoon

**Affiliations:** 1 Department of Molecular Science and Technology, Ajou University, Suwon 16499, Korea; moogoosla@ajou.ac.kr (H.J.C.); Han.Yong@mayo.edu (Y.D.H.); ympark@nnfc.re.kr (Y.M.P.); kkr4649@ajou.ac.kr (K.R.K.); 2 Nano-bio Application Team, National NanoFab Center (NNFC), Daejeon 34141, Korea; sjlee@nnfc.re.kr

**Keywords:** point-of-care testing, soft material-based channel, PDMS optical filter, smartphone-based biosensor, chromogenic biochemical assay, naked-eye detection

## Abstract

To overcome the time and space constraints in disease diagnosis via the biosensing approach, we developed a new signal-transducing strategy that can be applied to colorimetric optical biosensors. Our study is focused on implementation of a signal transduction technology that can directly translate the color intensity signals—that require complicated optical equipment for the analysis—into signals that can be easily counted with the naked eye. Based on the selective light absorption and wavelength-filtering principles, our new optical signaling transducer was built from a common computer monitor and a smartphone. In this signal transducer, the liquid crystal display (LCD) panel of the computer monitor served as a light source and a signal guide generator. In addition, the smartphone was used as an optical receiver and signal display. As a biorecognition layer, a transparent and soft material-based biosensing channel was employed generating blue output via a target-specific bienzymatic chromogenic reaction. Using graphics editor software, we displayed the optical signal guide patterns containing multiple polygons (a triangle, circle, pentagon, heptagon, and 3/4 circle, each associated with a specified color ratio) on the LCD monitor panel. During observation of signal guide patterns displayed on the LCD monitor panel using a smartphone camera via the target analyte-loaded biosensing channel as a color-filtering layer, the number of observed polygons changed according to the concentration of the target analyte via the spectral correlation between absorbance changes in a solution of the biosensing channel and color emission properties of each type of polygon. By simple counting of the changes in the number of polygons registered by the smartphone camera, we could efficiently measure the concentration of a target analyte in a sample without complicated and expensive optical instruments. In a demonstration test on glucose as a model analyte, we could easily measure the concentration of glucose in the range from 0 to 10 mM.

## 1. Introduction

**Figure 1:**
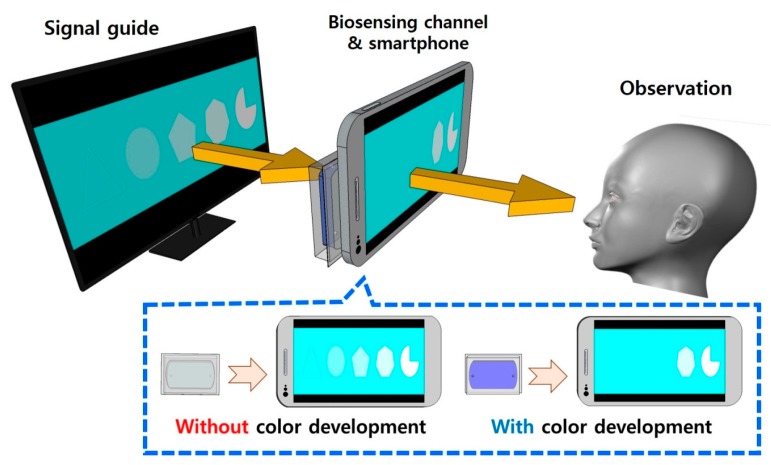
The scheme of the smartphone-based optical biosensing system. The numbers of signal guides on the screen were changed by superimposing a chromogen-containing biosensing channel. In the absence of a chromogen in the biosensing channel, intact clear-cut signal guides were observed, but the signal guides disappeared in the biosensing channel containing a blue product.

In the in vitro diagnostic (IVD) industry, development and commercialization of the biosensing technology enabling disease diagnosis wherever the patient is located has been regarded as the ideal goal. Therefore, the achievement of high portability, cost-effectiveness, and user-friendliness are considered major aims for robust analytical performance in biosensor technology research. Currently, due to good analytical performance including high accuracy and signal-to-noise ratio, optical biosensing technologies such as fluorescence analysis [1,2,3], surface plasmon resonance–based affinity biosensing [4,5,6], and ultraviolet/visible (UV/Vis) spectrophotometric biosensing are extensively investigated as promising approaches to the realization of point-of-care (POC) diagnostics. Because the robust analytical performance of these optical biosensing technologies can be attained only by means of a specific optical-signal-transducing technology (which has several drawbacks including low cost-effectiveness, high complexity, high power consumption, low portability, and poor user-friendliness), the applications of these conventional optical biosensing technologies to POC diagnostics are limited [7,8,9,10]. Thus, securing and developing an optical-signal-transducing system that can overcome those limitations on conventional transducers are regarded as the cornerstone for the development of a user-friendly POC optical biosensor. In this context, there is a growing research trend toward replacing conventional signal-transducing equipment with commercialized high-tech electronic devices such as scanners, optical storage devices, and smartphones, which provide low costs, low power consumption, and ease of use [11,12,13,14,15,16,17]. In this study, we focused on the development of a new optical-signal-transducing method that can replace the conventional spectrophotometry-based signal transduction of a colorimetric biosensor, by utilizing a common liquid crystal display (LCD) panel and a smartphone. The conventional spectrophotometric signal transducers employ a halogen lamp and monochromator as light source units at a specific wavelength. Because they require complicated configuration of optical components such as the prism and mirror, this system has poor portability, low cost-effectiveness, and substantial power consumption. In the case of an optical receiver of a conventional colorimetric transducer, the photodiode array is widely used. Unfortunately, it is also expensive and requires a complicated electric circuit to operate. Because those light sources and the optical receiver units are the most essential components of the optical signal transducer, the realization of a POC optical biosensor would depend on the introduction of a novel light source and optical receiver components (having high cost-effectiveness, user-friendliness, portability, and good optical properties at low power consumption) into the optical transducing system. To this end, we focused on the LCD panel of a computer monitor and smartphone, which are commercialized electronic products that humans always face in everyday life, as a light source unit and optical receiver unit, respectively. The LCD panel provides well-defined visible light whose color can be easily manipulated by controlling their color pixels (red, green, and blue ones). Compared to a monochromator, the LCD panel is already distributed to the public, and its control of brightness and color levels is easy. As an optical receiver, the smartphone provides a digital camera that allows for high-definition imaging. Besides, by means of its inherent display panel and the embedded image software, in situ analysis of a registered image can be accomplished on the smartphone. According to these features, we constructed a novel optical transducing system by reassembling a smartphone and LCD monitor (Figure 1). 

Into the newly developed transducing system, we introduced a horseradish peroxidase (HRP)-mediated chromogenic assay as a biosensing strategy. Conventionally, in the newly designed assay, the biorecognition reaction between the bioreceptor and target analyte induces color development under the action of HRP, and intensity of the resulting color development is affected by the concentration of the target analyte. The conventional colorimetric biosensors analyze changes in the color intensity according to the biochemical reaction to quantify the concentration of a target analyte. Nevertheless, measuring the color intensity or absorbance requires external analysis algorithms and specific software that is not user-friendly. To overcome this limitation in the conventional colorimetric biosensors, more intuitive and user-friendly signal quantification method—that can be operated without specific software—should be devised. To this end, we hypothesized that if the changes in color intensity can be converted into a change in a certain numerical parameter, a more convenient and intuitive quantitative analysis for the target analyte would be created. To realize this idea, we introduce the principle of a “secret message card”. The latter consists of a card containing message words and is covered by randomly painted patterns with various colors and a colored semitransparent cellophane film to read the message words without the interference from randomly painted color patterns on it. When the message words on the secret card are observed through the colored cellophane film, the patterns of the words with the same color as that of the cellophane film will be filtered out and disappear. This is because the spectrum of color light from the pattern overlaps with the absorption spectrum of the colored cellophane film. In contrast, the message words in a color different from that of the cellophane film pass through the cellophane film as is, and reach the observer, because there is no spectral overlap between the light of words and cellophane film. In this phenomenon, the colored cellophane film acts as an optical filter interfering with and filtering colored light. We attempted to apply this color-filtering principle of the secret message card to our optical transducing system as a signal quantification strategy. In this study, the colored cellophane film was replaced by a transparent and soft material-based biosensing channel which can induce development of the color (blue) via the aforementioned HRP-mediated chromogenic biochemical assay. To apply a biosensing channel for developed sensing system, the biosensing channel should be made of a transparent and soft material that can transmit the light. The silicone elastomer, such as Sylgard, Ecoflex, and Silbione, satisfies above condition, and it has high flexibility and stretchability so that they can be used as optical filters in a variety situation. In addition to this, the message words were replaced by the signal guide pattern containing polygonal figures with specific colors (a triangle, circle, pentagon, heptagon, and 3/4 circle). The signal guide was designed and produced in drawing software and was displayed on the LCD monitor, which utilizes (as a light source) equipment in our transducing system. On the LCD monitor panel, five polygon images, containing lights of red, green, and blue, were displayed as signal guide patterns. The area surrounding pattern images contains only green and blue light at the same ratio, consistent with the figure inside. In the biosensing channel, the HRP-mediated chromogenic biochemical assays were conducted to obtain blue reactants. Then, the resulting biosensing channel was mounted on the smartphone camera. When we observe polygons on the LCD monitor though this camera, the color of signal guide patterns is harmonized with the surrounding color because the red color in the pattern is filtered by the blue reactants in the biosensing channel as in the secret message card. Consequently, the number of visible polygons on the monitor would be changed by the extent of the chromogenic reaction in a biosensing channel. After counting of the polygons, the concentration of target analytes could be easily measured. In this study, a glucose assay was devised by means of HRP and chromogenic substrates. The details of the biosensing principle with analytical approaches are reported herein.

## 2. Materials and Methods 

### 2.1. Materials and Instruments

The Sylgard 184 silicone elastomer kit was acquired from Dow Corning (Midland, MI, USA). HRP was purchased from Toyobo (Osaka, Japan). Dopamine hydrochloride, glucose oxidase (GOx) from *Aspergillus niger*, 4-aminoantipyrine (4-AAP), amine-terminated G4 polyamidoamine (PAMAM) dendrimer, and Tris(hydroxymethyl)aminomethane (Tris) were acquired from Sigma-Aldrich (St. Louis, MO, USA), while *N*-Ethyl-*N*-(2-hydroxy-3-sulfopropyl)-3,5-dimethylaniline (MAOS) was from Dojindo (Kumamoto, Japan). Zoom Imaging Lens (PMAG 0.7X-4.5X) and CMOS Color USB Camera (resolution of 5.0 MP (megapixels)) were purchased from Edmund optics (Barrington, NJ, USA). To show the optical signal guide, LG computer monitor was used. A camera from the LG G2 smartphone was employed to register the obtained images. A phosphate-buffered saline containing 0.1 M phosphate and 0.15 M NaCl (PBS, pH 7.2) was prepared in doubly distilled and deionized water (DDW). 

### 2.2. Surface Modification of the Biosensing Channel

The biosensing channel was fabricated by means of polydimethylsiloxane (PDMS) upper layer and polyethylene (PET) film bottom layer. For the casting of PDMS upper layer having dimensions of 20 mm in length, 5 mm in width, and 2 mm in depth, an acrylic mold was prepared by computer numerical machining process. A 10:1 (*v*/*v*) mixture of Sylgard 184 monomer and initiator was poured onto the acrylic mold and placed in a vacuum chamber for 30 min to remove bubbles from the PDMS mixture. Then PDMS-filled acrylic mold was incubated in an oven at 80 °C for 1 h to cure PDMS [18]. Next, the cured PDMS substrate was detached from the acrylic mold and washed with distilled water. The washed substrates were dried in an oven at 80 °C for 20 min. The prepared PDMS channel layer has in/out transport channels at both sides and circular reaction region at the center of channel. For the solution manipulation including injection and draining, two holes were made on both sides of the PDMS channel using a puncher. To make a complete fluidic channel as a bottom layer, a transparent PET film having an adhesive layer on a single side was attached to the PDMS upper channel layer. The volume of the newly prepared biosensing channel was circa 200 μL. To construct the biorecognition layer on the biosensing channel surface, a polydopamine layer was coated with PDMS and PET surface as an intermediate layer for subsequent chemical conjugation processes [19]. Because the polydopamine coatings have an ability to immobilize primary-amine- and/or thiol-containing molecules by covalent interactions such as imine bond and Michael addition, the polydopamine layer could serve as an initial intermediate layer at the following bioconjugation steps [20,21]. Briefly, 2 mg/mL dopamine hydrochloride was prepared in a 10 mM Tris-HCl buffer solution, and its pH was adjusted to 8.5. The resultant dopamine solution was immediately injected into the biosensing channels, and the channel was incubated for 16 h at room temperature in a darkroom. After coating with polydopamine, the biosensing channel was washed five times with an excess of DDW. The resultant polydopamine-coated biosensing channels were filled with DDW and stored at 4 °C until further biomolecular modification.

### 2.3. Fabrication of the Biorecognition Layer for Glucose Biosensing

In this study, a bienzymatic colorimetric glucose assay using glucose oxidase (GOx) and HRP was chosen as a model biochemical assay to assess the biosensing applicability of the newly developed optical transducing strategy. To construct the bioreceptor layer enabling bienzymatic glucose assay in the biosensing channel, the multiple enzyme layers consisting of glucose oxidase and HRP were immobilized inside the polydopamine-coated biosensing channel by the layer-by-layer (LBL) technique using the PAMAM dendrimer as a building block molecule [22]. First, a 0.5% (*w*/*v*) G4 PAMAM dendrimer aqueous solution was prepared in 0.1 M phosphate buffer. Then, the dendrimer solution was injected into the polydopamine-coated channels and incubated in the dark for 16 h at room temperature. During this procedure, PAMAM dendrimers were covalently immobilized on the polydopamine layer via imine bond formation between primary amine groups of the dendrimer and catechol moieties of the polydopamine layer. The dendrimer-decorated biosensing channel was rinsed with PBS three times and filled with a 20 mM ethanolamine solution for 15 min to block the unreacted functional moieties in the polydopamine layer. Because 64 primary amine groups were expressed on the surface of the PAMAM G4 dendrimer in a globular shape, many amine groups should be exposed on the dendrimer-treated polydopamine layer. To conjugate enzymes to the amine-exposed biosensing channel surface via a covalent bond, the carbohydrate moieties on GOx and HRP were oxidized and converted into aldehyde groups by means of 40 μM sodium periodate. To build an HRP layer, a 1 mg/mL periodate-oxidized HRP solution (PBS, pH 6.8) was injected into the dendrimer-decorated biosensing channel and allowed to react for 1 h. In this procedure, HRP is immobilized on the amine-terminated sensing surface via Schiff’s base formation between aldehyde groups of HRP and primary amine groups of the dendrimer on the channel surface. Next, the intermediate dendrimer layer was built by applying an aqueous PAMAM dendrimer solution (0.5%, PBS) to the HRP-coated channel for 1 h. To form the GOx layer, a 1 mg/mL periodate-oxidized GOx solution (PBS, pH 6.8) was applied to the dendrimer-treated biosensing channel and allowed to react for 1 h. As in the HRP conjugation, GOx was immobilized on the dendrimer layer via Schiff’s base formation. For the strong color development in the bienzymatic colorimetric glucose sensing method, processes of construction of this bienzyme layer consisting of the dendrimer, HRP, dendrimer, and GOx was repeated three times (Figure S1, Supplementary Materials). The resulting modified biosensing channel coated with triple bienzyme layers was filled with PBS and stored at 4 °C until use. 

### 2.4. Preparation of the Optical Signal Guide

In this study, the optical signal guide (to be used as a reference light signal and light source for wavelength filtering–based colorimetric biosensing) was prepared in a graphics editor (software, Microsoft PowerPoint 2016) and displayed on the LCD monitor panel. First, five different polygonal figures including a triangle, circle, pentagon, heptagon, and 3/4 circle were drawn in the graphics editor. Then, using the color editing option, which allows for adjustment of the intensity of red (R), green (G), and blue (B) color elements step by step from 0 to 255, all the inner and outer regions of the polygon were painted with a color which contains only G and B color element. After initial color adjustment, the intensity of the R color element inside each polygon was manipulated, meanwhile the intensity values of G and B elements were maintained (255 and 255, respectively). The intensities of the R color element inside the triangle, circle, pentagon, heptagon, and 3/4 circle were adjusted to 135, 165, 195, 225, and 255, respectively. Compared to R color element varied from 135 to 255 in each polygon’s interior, the intensities of G and B color elements was maintained at 255 everywhere on a signal guide pattern. Then, the color-adjusted signal guide pattern image was documented as an image file in PNG or TIFF format to minimize the loss of its information resulting from file compression. In the following experiments, the signal guide image files were loaded onto the LCD display panel of a computer or smartphone by means of image viewer software (Gallery application). 

### 2.5. Verification of the Optical Biosensing Principle

To test whether the selective light absorption phenomenon–based optical signal transduction works, a model study involving a smartphone LCD display and a microscope was carried out. First, the signal guide pattern was displayed on the smartphone LCD panel and the glucose biosensing channel was mounted onto the signal guide–displaying smartphone LCD panel. Then, a portable microscope having charge-coupled device (CCD) imaging sensor was vertically installed above the biosensing channel and was connected to the computer for real-time acquisition of magnified images of RGB pixels in signal guide patterns on the smartphone display. A 10 mM glucose solution was prepared in PBS as a glucose sample meanwhile PBS alone (0 mM glucose) was prepared and served as a control sample. As a chromogenic substrate for the enzymatic colorimetric glucose assay, Trinder’s reagent solution containing 20 mM 4-AAP and 2 mM MAOS was prepared in PBS. A 1:1 mixture of the glucose sample and chromogenic substrate solution was injected into the smartphone-mounted glucose biosensing channel. According to the progress of the glucose-mediated cascade enzyme reaction, the colorless solution inside biosensing channel gradually turned into blue. In this setup, the image of RGB pixels on the smartphone LCD panel passes through the biosensing channel on top of it and reaches the microscope imaging sensor. In this process, the light of each RGB pixel is partially absorbed by the blue end product inside the glucose biosensing channel, and thus the light intensity of each RGB pixel observed by the microscope changes. After 5 min of the reaction, images of signal guide patterns and each RGB pixel were documented as image files. Finally, the color intensity of signal guide pattern and pixel images was quantitatively analyzed in NIH ImageJ software (Version: 1.50i)

### 2.6. Glucose Analysis by Enzymatic Colorimetric Assay

To demonstrate the applicability of the newly developed signal transducing technology to point-of-care biosensing, quantitative glucose detection (using a smartphone and a computer LCD monitor panel) was accomplished. First, glucose samples at various concentrations (0, 1.25, 2.5, 5 and 10 mM) were prepared. As a chromogenic substrate, Trinder’s reagent solution containing 20 mM 4-AAP and 2 mM MAOS was prepared. Right before the glucose assays, each glucose sample and Trinder’s reagent solution was mixed in a 1:1 volume ratio. The glucose biosensing channel, which is modified with triple bienzyme (GOx and HRP) layers was mounted on the smartphone camera lens using a clip. The signal guide image was displayed on the computer LCD monitor panel. Then, into the glucose-biosensing channel, the prepared glucose-chromogen mixture (solution) was injected. Under this setup, the signal guide patterns displayed on the LCD monitor panel can be observed and documented through the biosensing channel-mounted digital camera of a smartphone. As the glucose-mediated biocatalytic reaction proceeded, concentration of the blue assay end product inside the channel was increased and thereby its red-light absorbance was also increased. Under these circumstances, the number of remaining polygons in the digitally recorded signal guide image on smartphone camera may be changed by correlation between changes in red-light absorbance of glucose assay end product and the varied red intensities of each polygon. The image of the signal guide pattern was registered every 1 min during 5 min of total assay time. To minimize the signal interference from external light, all glucose detection tests were carried out under darkroom condition. After the image acquisition, the polygons in registered pattern images were counted with the naked eye and in the image analysis software (Pattern-recognition option in the Microsoft PowerPoint 2016).

## 3. Results

### 3.1. The Basic Signal-Transducing Principle: Spectral Correlation between the Chromogenic Solution and Color Pixels on the LCD Panel

In this study, we focused on the development of a new signal-transducing technology that can efficiently translate the colorimetric signal of a biochemical assay into signals that can be easily quantified by the naked eye. To convert the color intensity signal, which linearly depends on target analyte concentration, into visually countable signals without complicated optical instruments, here we employed the correlation of the absorption spectrum of the chromogenic compound and variations in RGB color pixel intensities on the computer LCD monitor panel as a major signal-transducing principle. 

**Figure 2:**
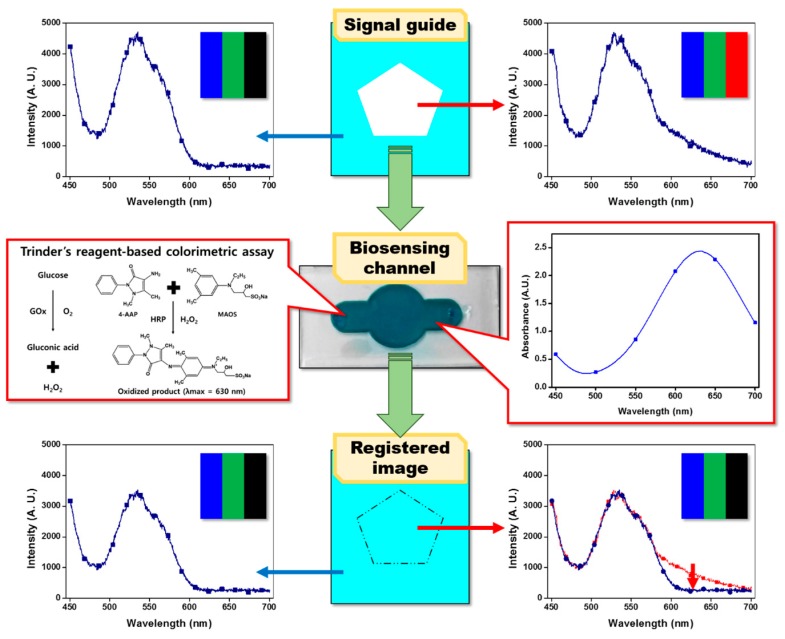
Illustration of the principles used in the newly developed optical biosensing system. The signal guide consists of red, green, and blue light at a certain ratio, and the area surrounding the polygons contains only green and blue light. After locating the biosensing channel that has the blue chromogen on the signal guide, we converted the color of signal guide into blue owing to absorption of the red light by chromogen.

As for the LCD monitor panel, it is composed of numerous tiny red (R), green (G), and blue (B) pixels and those R, G, and B pixels provide light with maximum intensity near 600, 530, and 400 nm, respectively. Because the light intensity of those RGB pixels can be quantitatively controlled step by step from 0 to 255 in a graphics editor, we can generate a light source of a specific wavelength by controlling RGB pixel intensities. Based on this feature, we introduced the LCD panel as a light source for a conventional chromogenic biochemical assay that requires light of a specific wavelength in the quantitative analysis of assay-derived colored end product. In this study, as a biochemical assay principle, Trinder’s reaction–based bienzymatic chromogenic glucose assay was evaluated [21,23,24]. In the presence of glucose, GOx generates H_2_O_2_, and the latter is catalytically degraded by HRP. Under the influence of H_2_O_2_ decomposition by HRP, colorless Trinder’s chromogenic substrate compounds (4-AAP and MAOS) are conjugated with each other and form a blue end product. This blue end product exhibits maximum absorbance at 630 nm (Figure 2, middle panel). Additionally, the amount of the blue reactant in the glucose assay and the intensity of its absorption spectrum are proportional to the concentration of glucose (Figure S2, Supplementary Materials). Considering that the light in the 630 nm region absorbed by the blue end product of the glucose assay is red light, a signal guide pattern (i.e., a set of polygons capable of presenting the change of red light according to the chromogenic reaction as a visualized information and providing it to the user) was designed as shown in Figure 2. Fundamentally, the signal guide pattern is a digital drawing of a polygon which is designed to have different color pixel characteristics inside and outside. The color intensity of each G and B color pixel inside and outside a polygon was adjusted to the same value of 255 in the graphics editor. For the R color pixel, the interior and surroundings of the polygon were set to 255 and 0, respectively, as shown in Figure 2 (upper panel). Because only green and blue colors were activated, the outside of the polygon had a cyan color and emission spectrum peaks near 400 and 530 nm. In contrast, the interior of the polygon showed white color because red, green, and blue lights are simultaneously emitted and mixed. Although the emission spectrum of the polygon’s inside area was similar to that outside the polygon, emission intensity near 600 nm corresponding to red light was increased as intended. When the light emitted from the signal guide pattern of a computer LCD panel was passed through the blue end product of the glucose assay (λ_max_ = 630 nm), the red-light portion was absorbed into the blue chromogenic compound while green and blue light were barely affected. Due to the red color filter-like effect of the chromogenic compound, it was observed that the white polygon changed to a cyan polygon during examination of the signal guide pattern on the LCD panel through the biosensing channel that turned blue (Figure 2, bottom panel). As shown in the emission spectrum in Figure 2 (bottom right panel), when light passed through the blue chromogenic compound, the intensity of the emission spectrum near 600 nm, acquired from the polygon inside, decreased and became similar to the emission spectrum outside the polygon. Conversely, when we see surroundings of a polygon in a signal guide pattern through the biosensing channel that developed a color, its cyan color and initial emission spectral property were retained because the light from the cyan region outside the polygon does not contain red light. When the concentration of glucose is high enough to generate the colored assay reactant in sufficient amounts that can absorb all red light, the red-color-filtering effect of the chromogenic compound induces synchronization of the colors inside and outside of a polygon observed through the biosensing channel, and thus makes the polygon disappear (Figure 2, bottom panel). Via this principle, changes in the colorimetric signal induced by a biochemical assay can be easily visualized as disappearance of a polygonal pattern, without complicated optical instruments. Nonetheless, in the present state, it can be used only for qualitative detection of glucose at a specific concentration. Therefore, to apply this basic signal transducing principle to quantitative glucose biosensing, the signal guide pattern was engineered and improved as follows.

### 3.2. The Principle of Quantitative Glucose Analysis by the New Signal-Transducing Method

**Figure 3:**
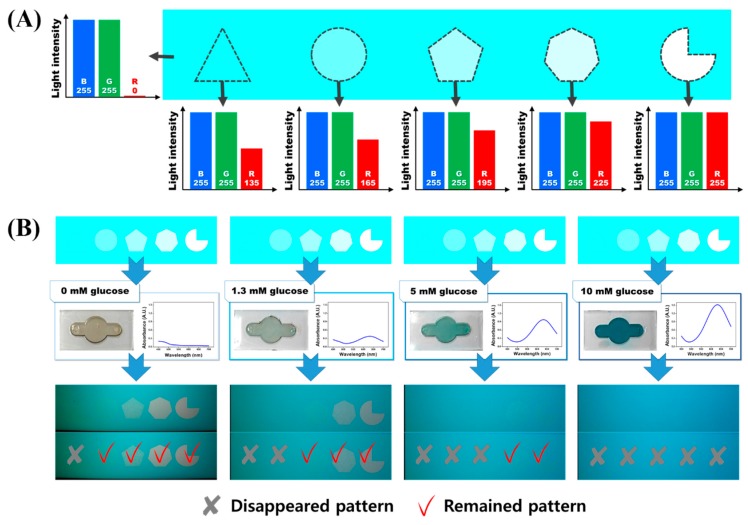
Fabrication of the signal guides on the LED display including the various intensity of red light: (**A**) The signal guides have a fixed intensity of blue and green light with varying intensity of red light, and the surrounding area contains blue and green of fixed intensity; and (**B**) the images of a signal guide during superposition of a biosensing channel. The numbers of signal guides change according to the chromogen concentration.

For application of our new optical-signal-transducing principle to quantitative glucose biosensing, a new signal guide pattern composed of five polygons, including a triangle, circle, pentagon, heptagon, and 3/4 circle was prepared (Figure 3A) and displayed on the computer LCD monitor panel. For G and B pixels, the inside and outside regions of each polygon were fixed at 255 as in the initial design. In contrast, the R pixel intensities within each polygon were gradually varied from 135 to 255 (135 for triangles, 165 for circles, 195 for pentagons, 225 for heptagons, and 255 for 3/4 circles). Because each polygon has different R pixel intensity, the glucose concentration for disappearance of each pattern should vary. In the case of a low glucose concentration, the amount of the blue reactant produced in the glucose assay is small. This low concentration of the colored product yields low red-light absorbance; therefore, polygons whose R color pixel intensity is low (e.g., triangle and/or circle) would disappear preferentially. By contrast, when the blue product is excessively produced at a high concentration of glucose in the sample being analyzed, most polygons would disappear because the almost red light from R pixels will be fully absorbed by the blue reactant of the assay. To assess the correlation between glucose concentration and disappearance of polygons having various R pixel intensities, we monitored the prepared polygons on the LCD monitor panel using a smartphone camera through a glucose-sensing channel loaded with a glucose sample (0, 1.3, 5, or 10 mM) for 5 min.

As shown in the middle panel of Figure 3B, numbers of polygons that disappeared increased according to the increase in glucose concentration. As the glucose concentration increases, intensities of color and absorption spectra of the glucose assay reactant increased (Figure 3B, middle panel). As shown in the bottom panel of Figure 3B, in an assay with 1.3 mM glucose, polygons with low R pixel intensity (triangle and circle) preferentially disappeared because the R pixel intensity was low enough to be fully absorbed by a small amount of the blue reactant of the assay. In contrast, polygons with relatively high R pixel intensity (pentagon, heptagon, and 3/4 circle) did not disappear because their red-light intensity exceeds the red-light absorption capacity of the blue reactant that was produced at a low concentration of glucose. (Note that the polygons that disappeared were marked with X and the remaining polygons were marked with V.). In the glucose assay applied to a 5 mM glucose sample, the pentagon disappeared, meanwhile heptagon and 3/4 circle remained. In a glucose assay with a 10 mM glucose sample, all polygons disappeared because the red-light absorption capacity of the reactant generated in the glucose assay was high enough to completely absorb all red light of R pixels in polygons. In this experiment, we found that the color intensity linearly changing with alterations in the target analyte concentration could be translated into visual signals that can be counted with the naked eye in this newly developed optical-signal-transducing technology. Using this principle, we can easily evaluate the concentration of glucose by counting the polygons that disappeared (or remained).

### 3.3. Validation of the Optical-Signal-Transducing Principle at a Pixel Level

**Figure 4:**
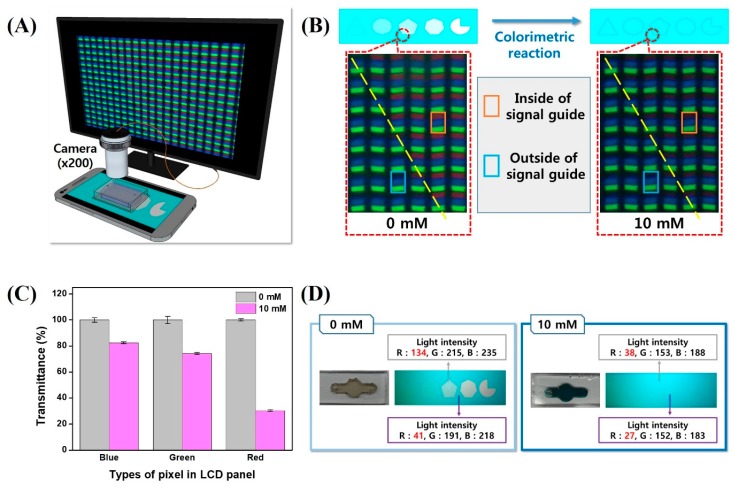
Alterations of RGB intensity on the mobile screen depending on the analysis of 0 or 10 mM glucose in the glucose assay: (**A**) The confirmation of changes in RGB pixels on the screen by microscopy. (**B**) The resulting images of the RGB pattern in the signal guide. The red light in the signal guide was changed by superimposing the blue chromogen-containing biosensing channel. (**C**) The transmittance ratio of blue, green, and red light in the signal guides. The blue and green lights slightly changed, while the red light is significantly altered. (**D**) The resulting images of a whole signal guide. The numbers of signal guides changed in the presence of a chromogen, and the red light highly decreased in the glucose assay involving a 10 mM glucose sample.

To test whether the conversion of color intensity into a visually assessable signal was accomplished by the spectral correlation between the absorption spectrum of the chromogenic compound and light intensity of R pixels on the LCD panel displaying the signal guide pattern, a microscopic analysis was conducted. For this experiment, the signal guide pattern image (polygons) was loaded and displayed on the smartphone LCD panel instead of the computer LCD monitor panel. In the polygon-displaying region of the smartphone LCD panel, the glucose biosensing channels, containing a mixture of glucose samples (0 and 10 mM) and Trinder’s chromogenic reagents, were mounted. After the compounds were allowed to react for 5 min, the RGB color pixels in the signal guide pattern of the LCD panel were monitored using a portable microscope with a CCD digital camera (Figure 4A).

Figure 4B shows the magnified pixel images of the smartphone LCD panel where the polygons were displayed. The yellow dashed line indicates the boundary between the inside and outside of a pentagon. Regarding an assay result for 0 mM glucose (left panel), only G and B color pixels were observed in the region outside the pentagon as initially adjusted by means of the graphics software (Microsoft PowerPoint 2016). Inside the pentagon, all RGB color pixels were observed, just as in the initial design because the blue end product (of the glucose assay) absorbing red light was not generated in the absence of glucose. Because the differences in R pixel intensities between inside and outside regions of the pentagon persisted after the glucose assay of the 0 mM glucose sample, the pentagon shape did not disappear and was visible as initially designed. Regarding the assay result for a 10 mM glucose sample (Figure 4B, right panel), G and R pixels in the regions inside and outside the pentagon retained their light intensity even after the intense color development. Nevertheless, the intensity of the R pixels inside the pentagon sharply decreased and was barely distinguishable visually. As a result, the image of the R pixel inside the pentagon at the reduced light intensity became similar to the image of the R pixel outside the pentagon. In the obtained findings, readers can see that the disappearance of the pentagon pattern in the course of the chromogenic glucose assay reaction is closely related to the spectral correlation of R pixel intensity and the intensity of the absorption spectrum of the glucose assay reactant. To confirm this result quantitatively, the transmittance of light from RGB pixels of the signal guide pattern (pentagon) that passed through the glucose assay reactant (10 mM glucose) was calculated by analyzing color intensity of RGB pixels in the registered image by means of the NIH ImageJ software. The relative transmittance of light from RGB pixels of the pentagon pattern that passed through 10 mM glucose was calculated based on the intensity of light (from RGB pixels inside the pentagon) that passed through the 0 mM glucose reactant, which was set to 100%. As shown in Figure 4C, the transmittance for R, G, and B pixels inside the pentagon (toward the reactant corresponding to the 10 mM glucose sample) decreased by 70%, 25%, and 17%, respectively, relative to reference transmittance (0 mM glucose).

Additionally, the color synchronization of the signal guide pattern according to the progress of the chromogenic reaction in the glucose assay was quantitatively analyzed from a macroscopic view. The image of the signal guide pattern was registered using a smartphone camera through glucose-sensing channel loaded with the glucose sample (0 or 10 mM). By means of the registered images, the RGB color intensities inside and outside the pentagon were extracted and quantitatively analyzed in the NIH ImageJ software, as shown in Figure 4D. In this analysis, the image of the signal guide pattern that was photographed by the smartphone camera served as a model of how color synchronization of patterns looks to the naked eye. As shown in the left panel of Figure 4D, in the glucose assay applied to the 0 mM glucose sample, the intensity of RGB color elements inside the pentagon was measured and found to be 134 (red), 215 (green), and 235 (blue). Besides, the intensity of RGB color elements outside the pentagon was 41 (red), 191 (green), and 218 (blue), respectively. Because of the differences in intensity of red color elements between inside and outside regions of the pentagon, the shape and color of the pentagon were easily distinguishable from the cyan surrounding area when 0 mM glucose was tested in the assay. In contrast, as shown in the right panel of Figure 4D, the intensities of RGB color elements inside the pentagon were registered at 38 (red), 153 (green), and 188 (blue). These data are similar to RGB color intensity values outside the pattern (27 for red, 152 for green, and 183 for blue). Because the RGB color profiles inside and outside the pentagon were similar, the shape and color of pentagon were indistinguishable from the cyan surrounding area to the naked eye when 10 mM glucose was tested in the glucose assay. From the obtained results, we concluded that the newly designed optical-signal-transducing technique that translates the color intensity into the visually assessable signal is based on the spectral correlation of the absorption spectra of chromogenic compounds and the light intensity of R pixels on the signal guide patterns of the LCD panel.

### 3.4. Optimization of Conditions for Glucose Biosensing Based on the New Transducing Technology

In the newly developed optical-signal-transducing technique, the increase in color intensity and absorbance of the reactant in the glucose assay is converted into an increased number of polygons that disappeared. Therefore, for the accurate glucose biosensing using this transducing principle, the disappearance of a polygon should sensitively reflect a change in the concentration of glucose. Given that the disappearance of the polygon is directly related to the absorbance of the reactant in the glucose assay, the yield of the bienzyme-mediated chromogenic reaction should be improved for the sensitive glucose sensing. In this context, the amount of immobilized enzyme and the reaction time of a glucose assay (which are closely related to the production rate of the blue chromogenic reactant in the glucose assay) were optimized.

As a bioreceptor layer for the glucose biosensing, in this study, GOx and HRP were covalently immobilized on the surface of a biosensing channel via the LBL technique. The advantage of this technique for the construction of the enzyme-based biosensing interface is that the amount of enzymes to be immobilized on the biosensing surface can be adjusted by controlling the number of enzyme layers. Based on this feature, to determine the optimal amount of enzyme immobilization that would allow for a sensitive chromogenic reaction in the glucose assay, three enzyme surfaces with different numbers of enzyme layers were constructed and compared. As shown in Figure S1, HRP and GOx were alternately immobilized on the surface of a biosensing channel by means of the polydopamine technique and dendrimer. This bienzyme layer (BEL), composed of the dendrimer, HRP, and GOx, was regarded as a surface modification unit. By repeating the BEL construction procedures (Figure S1), single, double, and triple BEL layers (BEL1, BEL2, and BEL3, respectively) were built on the surface of the glucose-biosensing channel. To the prepared three types of glucose biosensing channel, a mixture of glucose (2.5 mM) and Trinder’s reagent was applied. Then, through the glucose-sensing channel loaded with a glucose sample (0 and 10 mM), the image of signal guide pattern on computer LCD monitor was observed and registered using a smartphone camera every 1 min up to 10 min.

**Figure 5:**
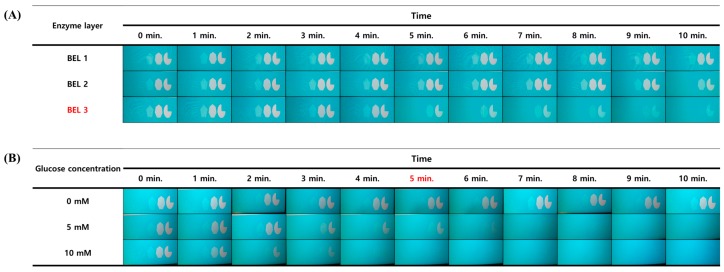
The resulting images of the optimization test for biosensing of surface modifications: (**A**) A 2.5 mM sample of glucose is assayed by means of the GOx/HRP catalysis. The one, two, and three enzyme layers were compared, and the images with color development were registered every 1 min up to 10 min. As the immobilized enzyme layer increases, the signal guides significantly changed. (**B**) The result of testing the reaction time by means of the three-enzyme layer. With reaction time at 5 min, the signal guides are clearly distinguished in accordance with the applied glucose concentration.

As shown in Figure 5A, in the assay involving BEL1, the distinguishable disappearance of polygons in the signal guide pattern was not observed for assay duration of 10 min. In the assay using BEL2, a polygon disappearance was observed at 9 min after the observation. These results indicate that the amount of enzymes in channels BEL1 and BEL2 was insufficient to generate blue end product absorbing red light of the signal guide pattern. In contrast, in the assay using BEL3, obvious polygon disappearance was observed 4 min earlier than for BEL2 (at 5 min after the start of observation). In addition, during the monitoring of the signal guide pattern through the BEL3 channel, the number of polygons that disappeared gradually increased with time, and finally only one polygon (3/4 circle) remained at 10 min after the start of observation. This result indicates that the amount of enzymes that is immobilized on the channel surface in the triple BEL format by the LBL method is sufficient to induce an intense color-development reaction that can effectively absorb red light of the signal guide patterns on the LCD panel. Therefore, BEL3 was chosen as a condition for the enzyme immobilization on the glucose-biosensing channel.

Next, the reaction duration for the glucose assay was optimized as follows, so that the assay can show the most distinguishable differences in disappearance of polygons between glucose concentrations. Into the BEL3-containing glucose biosensing channel, glucose samples at different glucose concentrations (0, 5, and 10 mM) was injected with Trinder’s reagent. Then, through the prepared glucose sensing channel, the image of the signal guide pattern on the LCD panel was monitored and registered using the smartphone camera every minute up to 10 min.

As shown in Figure 5B, when the reaction time was less than 5 min, notable differences in polygon disappearance among glucose concentrations were not detected. This finding indicates that the given reaction time (less than 5 min) is not enough to produce sufficient amount of the blue reactant in the glucose assay. In contrast, at 5 min of glucose assay time, the numbers of invisible polygons proportionally increased with the glucose concentration and those changes could be distinctively detected by the naked eye. When the reaction time exceeded 5 min, the number of invisible polygons in the glucose assay with the 5 mM glucose sample and that with 10 mM glucose was similar, and it was hard to distinguish their differences with the naked eye. This is because the blue reactant in the glucose assay was excessively produced at reaction duration over 5 min, and the excessively produced chromogenic compounds absorb most of red light emitted by the signal guide pattern. Based on this finding, reaction time of 5 min, which yielded clear-cut differences in the number of invisible polygons in accordance with the changes in glucose concentration, was selected as the optimal reaction time condition for quantitative glucose biosensing. The optimal reaction conditions derived from this experiment (BEL3-containing glucose biosensing channel and reaction time of 5 min) were applied to the following glucose-biosensing procedure.

### 3.5. LCD Panel-Based Glucose Detection by the Naked Eye

**Figure 6:**
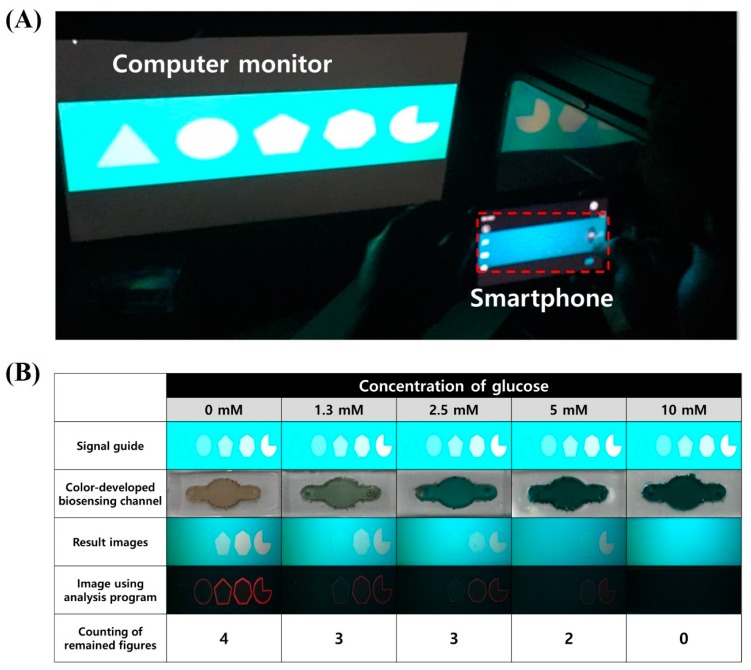
The glucose analysis based on the newly developed biosensing principle: (**A**) an image of application of the new biosensing system to a computer monitor; and (**B**) the resulting images of biochemical reactions at various glucose concentrations in the sample (0, 1.3, 2.5, 5, or 10 mM) after GOx/HRP catalysis. The smartphone-based quantitative analysis was conducted by naked-eye observation, and computational calculation was implemented to provide accurate counting.

To demonstrate the applicability of the newly developed optical transducing technology to practical biosensing, a quantitative glucose assay was carried out. On the LCD panel of the computer monitor, the signal guide pattern (Figure 3) was displayed. To the BEL3-modified glucose biosensing channel, a mixture of a glucose sample at various concentrations (0, 1.3, 2.5, 5, and 10 mM) and Trinder’s reagent was applied. The glucose-loaded biosensing channel was mounted on the smartphone camera lens and was incubated for 5 min for color development. After that, as depicted in Figure 6A, the image of signal guide patterns on the LCD panel was observed and documented by the smartphone camera, with the glucose-biosensing channel that developed blue color serving as a color filter. Finally, the remaining polygons in the registered signal guide image were counted with the naked eye. Simultaneously, the remaining polygons in the signal guide pattern were also counted by the pattern-recognition option of the graphics editor (software, Microsoft PowerPoint 2016) to compare the counting results between the naked-eye analysis and algorithmic analysis. The resulting images and the polygon counting results are shown in Figure 6B.

As expected, depending on the increase in glucose concentration, the color intensity of the glucose assay reactant in the biosensing channel increased, and the number of remaining polygons in the observed signal guide pattern decreased. In this experiment, when the concentration of the blue glucose analyte in the biosensing channel increased, the absorption of red light, emitted inside the polygon in the signal guide pattern, was enhanced. This red-light absorption induced synchronization of the colors inside and outside the polygon, so that the polygon disappears in a given observation condition. In the glucose assay with low concentrations of the glucose sample (0, 1.3, and 2.5 mM), the triangle, circle, and pentagon disappeared in this order because the intensity of their R pixel was weak enough to be fully absorbed by the reactant in the glucose assay at low concentrations (135 for the triangle; 165 for circle; 195 for pentagon). When those polygons showing low R pixel intensity disappeared at low glucose concentrations, the heptagon and 3/4 circle (yielding high R pixel intensity) were still visible. The polygons that have high R pixel intensity such as the heptagon (R: 225) and 3/4 circle (R: 255) disappeared only at the high glucose concentrations (5 and 10 mM). The obtained results revealed that the disappearance of the polygons was dependent on the spectral correlation of absorbance of the glucose assay reactant and emission intensity of R pixels in polygons as we have initially intended.

To demonstrate that the naked-eye counting results were not affected by the subjective intervention of individual observers, the obtained naked-eye results were compared with the algorithmic analysis results. In the naked-eye analysis, the number of residual polygons observed at 0, 1.3, 2.5, 5, and 10 mM glucose in the samples was 4, 3, 3, 2, and 0, respectively. As shown in Figure 6B, the residual polygons which were recognized and detected by the pattern recognition algorithm were outlined in red color. As a result, the number of red polygons was the same as the number of remaining polygons that were counted by the naked eye. This result indicates that the newly designed optical transducing technique is a promising approach to implementing a user-friendly biosensing system based on convenient naked-eye analysis. 

In our glucose-biosensing results, the numbers of remaining polygons at 1.3 mM glucose in the samples were the same as those at 2.5 mM glucose. Strictly speaking, this result means that the newly developed glucose-biosensing method is not suitable for detection of low concentrations of glucose. Nevertheless, considering that the diagnostic criterion for a glycemic profile to indicate diabetes mellitus is 5 mM glucose, we expect that our glucose-biosensing technique based on the new optical transducing technology can be used for the screening of diabetic patients and healthy people. 

From the obtained findings, we concluded that the newly developed biosensing method based on the new optical-signal-transducing principle utilizing spectral correlation between the absorption spectrum of a chromogenic reactant and color profiles of pixels in the LCD panel for glucose will help to realize a POC diagnostic system that is highly user-friendly and cost-effective.

## 4. Conclusions

By combining the LCD display panel, smartphone, transparent and soft material-based optical filter, and enzymatic colorimetric assay principle, we developed a new optical-signal-transducing technology. The LCD panel that contains numerous color pixels whose intensity can be easily regulated by software served as a light source in the new signal transducing system. The colored end product of the enzymatic chromogenic assay was used to interfere with the RGB lights inside and outside a polygon on the LCD panel based on the color filter-like effect. By means of the spectral correlation between the absorption spectrum of the colorimetric reactant under study and color profiles of pixels on the LCD panel, the color intensity signals that change linearly with the alterations in analyte concentration were successfully converted to the visual signal (e.g., disappearance of a polygon) that can be counted as an integer number by the naked eye. Using the new signal-transducing system, we could achieve biosensing of glucose in the range of 0–10 mM, covering the clinical criteria for diabetes mellitus screening, without complicated optical instruments. Because a common LCD monitor panel and a smartphone were used as optical instruments, the newly developed signal-transducing system provides high user-friendliness and operational convenience. Besides, because these devices are widely distributed in the population nowadays, the newly developed system should be helpful for implementation of a POC biosensing system overcoming the constraints in time and space on diagnostics. Additionally, given that the HRP-mediated chromogenic reaction served as a principle of colorimetric signal generation, the proposed optical-signal-transducing system can be applied to the HRP-mediated affinity biosensing such as an enzyme-linked immunosorbent assay involving HRP as a signaling reporter. Judging by the obtained results and considerations, we expect that this optical transducing approach will provide insights into materialization of a POC biosensing system that is user-friendly and has a wide application range.

## Supplementary Materials

The following are available online at http://www.mdpi.com/1996-1944/11/3/388/s1. Figure S1: The workflow of the manufacture of a biorecognition layer on the biosensing channel surface. Construction of the enzyme layer for detection of glucose by means of GOx, HRP, and a dendrimer; Figure S2: Changes in the absorbance at visible wavelengths caused by various concentrations of glucose in the sample.
