# Supplementary material for: An Optical Biosensing Strategy Based on Selective Light Absorption and Wavelength Filtering from Chromogenic Reaction
Source: Materials (Basel). 2018 Mar 6;11(3):388. doi: 10.3390/ma11030388 (PMC5872967; doi:10.3390/ma11030388)
Supplement: Supplementary file 1 [file materials-11-00388-s001.pdf]

Supplementary information

An optical biosensing strategy based on selective light absorption and wavelength filtering from chromogenic reaction

Hyeong Jin Chun[1], Yong Duk Han[1], Yoo Min Park[1,2], Ka Ram Kim[1], Seok Jae Lee[2], and Hyun C. Yoon[1]*

[1] *Department of Molecular Science and Technology, Ajou University, Suwon 16499, South Korea*

[2] *Nanobio Application Team, National NanoFab Center (NNFC), Daejeon 34141, South Korea*

* Author to whom correspondence should be addressed:

E-mail: hcyoon@ajou.ac.kr

Supporting information

Figure S1

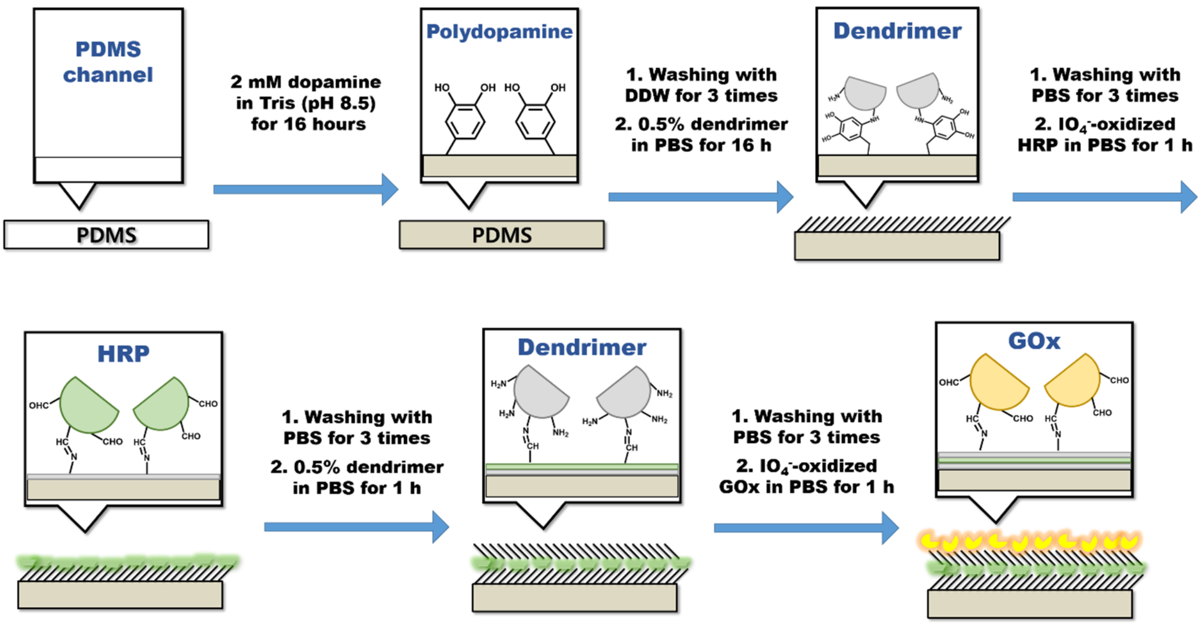

Figure S1. The workflow of the manufacture of a biorecognition layer on the biosensing channel surface. Construction of the enzyme layer for detection of glucose by means of GOx, HRP, and a dendrimer.

Figure S2

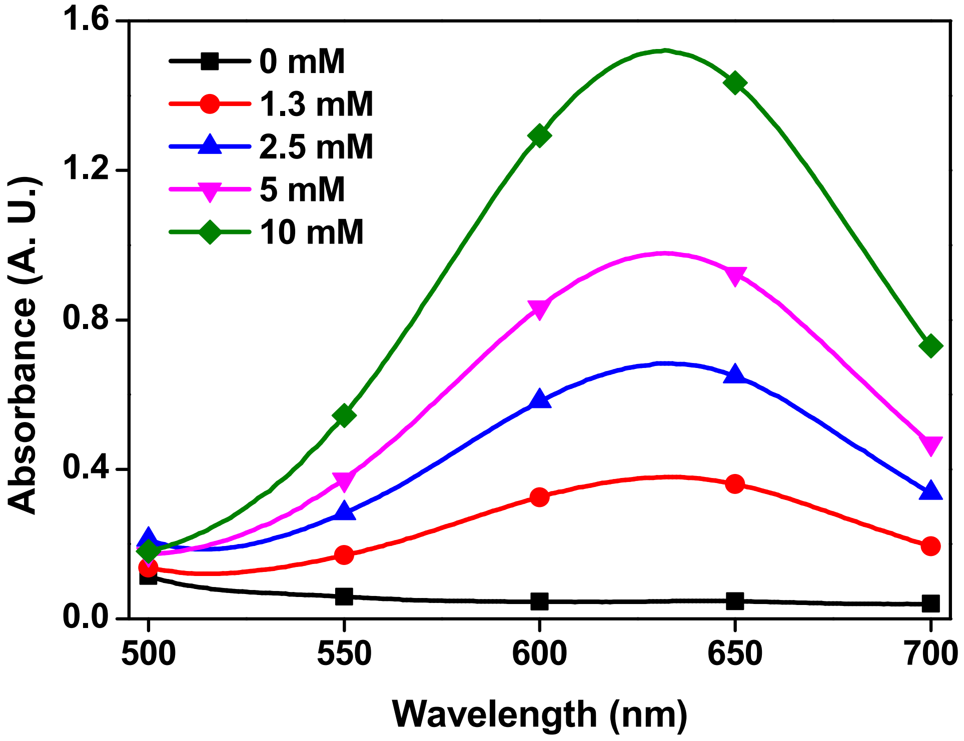

Figure S2. Changes in the absorbance at visible wavelengths caused by various concentrations of glucose in the sample (0, 1.3, 2.5, 5, or 10 mM).
